# Review on the Traction System Sensor Technology of a Rail Transit Train

**DOI:** 10.3390/s17061356

**Published:** 2017-06-11

**Authors:** Jianghua Feng, Junfeng Xu, Wu Liao, Yong Liu

**Affiliations:** Research Institute of Electrical Technology & Material Engineering, CRRC Zhuzhou Institute Company, Ltd., Zhuzhou 412001, China; xujf@csrzic.com (J.X.); liaowu@csrzic.com (W.L.); liuyong3@csrzic.com (Y.L.)

**Keywords:** traction system, sensor, signal processing, fault diagnosis, intelligent sensor

## Abstract

The development of high-speed intelligent rail transit has increased the number of sensors applied on trains. These play an important role in train state control and monitoring. These sensors generally work in a severe environment, so the key problem for sensor data acquisition is to ensure data accuracy and reliability. In this paper, we follow the sequence of sensor signal flow, present sensor signal sensing technology, sensor data acquisition, and processing technology, as well as sensor fault diagnosis technology based on the voltage, current, speed, and temperature sensors which are commonly used in train traction systems. Finally, intelligent sensors and future research directions of rail transit train sensors are discussed.

## 1. Introduction

Rail transit has become a widely utilized means of transportation [1,2] owing to its large capacity, high speed, low energy consumption, and other outstanding characteristics. Since the *Medium- and Long-Term Railway Network Plan* was proposed in 2004, China has made remarkable achievements in high-speed rail development. So far, high speed railway operation mileage has exceeded 20,000 km, which accounts for more than 60% of global operation mileage. China has become the country with the fastest development of high speed railway in the world, with the strongest integration capacity, longest operation mileage, fastest operation speed and the largest scale of construction [3,4]. The rapid development of high-speed trains cannot depart from the support of high performance sensor technology. As a type of detector, sensors can feel measured information and convert it into electrical signals according to certain rules; this plays an indispensable role in ensuring the performance, safety, and intelligence of rail transit train systems. In terms of the high performance control of the traction drive system, the advanced Alternating Current (AC) drive control technology, such as the direct torque control and vector control of the traction motor, is inseparable from the sensor for testing the states of traction motors and inverters [5,6,7]; in terms of the safety of the train operation, the sensors play an important role in detecting the system state; in terms of intelligence of the rail transit, how to grasp more data for train operation to support the intelligent control of the train. Intelligent monitoring and diagnosis, intelligent maintenance, and other relevant functions are also inseparable from the advanced sensors for monitoring and controlling the train [8,9]. The CRH380AL for example, is equipped with more than 1000 sensors [10]. 

The current study takes the train traction system sensor as an example. A traction system mainly consists of a transformer, AC/direct current (DC) rectifier, inverter, and traction motor. In this paper, we follow the sequence of the sensor signal flow, consequently present the sensor signal sensing technology, and the sensor data acquisition and processing technology, as well as the sensor fault diagnosis technology, based on voltage, current, speed and temperature sensors which are commonly used in the train traction system in Section 2, Section 3, and Section 4 respectively. In Section 5, we have analyzed the characteristics and key technology of the intelligent sensors. Future research hotspots and challenges for the railway transit sensor are discussed in Section 6. Finally, conclusions are drawn in Section 7.

## 2. Signal Sensing Technologies of Traction System Sensors 

**Figure 1:**
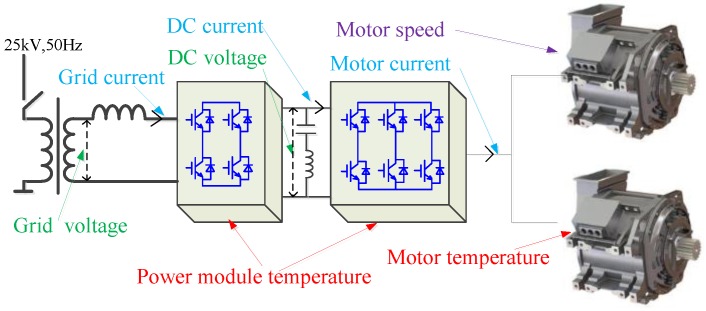
Traction system collected information.

Some important information that the train traction system needs to collect is shown in Figure 1, including grid voltage and current, traction motor current and temperature, power module temperature and motor speed, and other speed-related signals [11,12,13]. Thus, a variety of sensors are needed for sensing different signals, these commonly used sensors can be divided into four categories: current sensor, voltage sensor, speed sensor and temperature sensor. The most widely used signal sensing technologies of traction system sensors are introduced below.

### 2.1. Signal Sensing Principle of Current and Voltage Sensors

**Figure 2:**
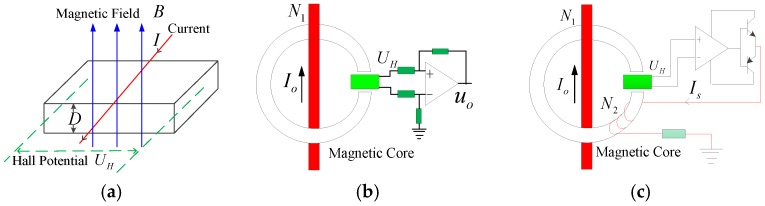
(**a**) Schematic diagram of the Hall-effect; (**b**) Schematic diagram of direct measurement by the Hall current sensor; (**c**) Schematic diagram of the zero-flux Hall current sensor.

As the most important electrical signals of traction systems, voltage and current signals serve as the basis to control the four-quadrant rectifier and traction inverter. Power electronic devices are extensively used on trains, and the measurement of the voltage and current satisfies such requirements as good dynamic performance, wide working bandwidth, and availability to measure any waveform. Hall sensors [14] can meet such requirements and are widely used on trains. The design of Hall current sensors are based on the Hall-effect, and the principle of this effect is presented as follows. As shown in Figure 2a, suppose the thickness and through current is *D* and *I* respectively for the Hall semiconductor, and suppose that a magnetic field exists whose magnetic flux density is *B*, located perpendicular to the surface of the Hall element; the magnetic field will generate a Hall electric potential perpendicular to the directions of the current and magnetic field. The scale of this potential is:

(1) $$U_{H}=R_{H}\frac{IB}{D}$$

where *R_H_* is the Hall coefficient of the Hall sensor. When the current through the Hall device is fixed, the Hall voltage *U_H_* is directly proportional to the magnetic field *B*. The Hall device can be employed to detect the magnetic field *B* induced by the measured current if *U_H_* is measured, then the current can be obtained according to the Ampere Circuital Theorem.

The actual Hall current sensors have two types of structures: direct measurement sensors (Figure 2b) and zero-flux sensors (Figure 2c) [15]. The direct measurement sensors have the advantage of consisting of a simple structure. However, the magnetic core may become saturated, and result in an eddy-current with hysteresis losses existing in the magnetic core. The zero-flux sensors can amplify the output voltage of the Hall device, output a compensation current *Is* through the compensation coil *N_2_*, and counteract the magnetic field generated from the measured current, so the magnetic flux density is basically zero in the magnetic core, and will not make the magnetic core become saturated or produce significant hysteresis and eddy-current loss. The relationship between the balanced output current and the primary coil current is:

(2) $$I_{o}=\frac{N_{2}}{N_{1}}I_{s}$$

The Hall voltage sensor is similar to the current sensor in working principle，but only converts voltage into current.

### 2.2. Signal Sensing Principle for Speed Sensors

Speed sensors are adopted to detect the traction motor speed. The speed sensors which are widely used on traction systems mainly include Hall speed sensors and photoelectric speed sensors.

**Figure 3:**
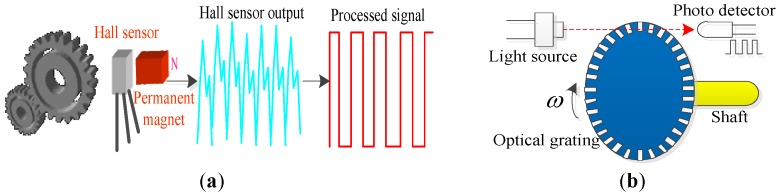
(**a**) Schematic diagram for the working principle of Hall speed sensors; (**b**) Schematic diagram for the principle of photoelectric speed sensors.

Hall speed sensors also utilize the Hall-effect, and its working principle is illustrated in Figure 3a. Hall speed sensors are usually used together with wheel gears, whose rotation can have the magnetic reluctance changing periodically along with the air gap. The Hall device outputs tiny pulse signals, and the rotation speed can be determined by means of processed pulse signals [16]. Hall speed sensors are widely used on trains by virtue of the output signals of square waves featuring high sensitivity, large output amplitude, small temperature drift, and other outstanding advantages.

The working principle of the photoelectric speed sensor is the photoelectric effect [17], as shown in Figure 3b. Photoelectric speed sensors change light signals into electrical impulses through optical grating, and finally convert this into the motor speed. Photoelectric speed sensors can output square wave signals with high precision, high resolution, and high reliability. However, the photoelectric components easily age. Thus, in the interests of safety, the photoelectric module must be replaced after the photoelectric sensor has been in operation for 300,000 km, or five years.

### 2.3. Signal Sensing Principle of Temperature Sensors

The temperature sensors applied in the traction system mainly include platinum resistance temperature sensors and negative temperature coefficient (NTC) thermistor temperature sensors.

The platinum resistance temperature sensor resistance changes with the temperature, and the function expression for PT100 is [18]:

(3) $$R_{T}=\left\{ \begin{array}{ll} R_{0}(1+AT+BT^{2}+C(T-100)T^{3} & -200{}^{\circ}C\leq T\leq 0{}^{\circ}C\\ R_{0}(1+AT+BT^{2}) & 0{}^{\circ}C\leq T\leq 600{}^{\circ}C \end{array}\right.$$

where *R_T_* and *R*_0_ indicate the platinum resistance values at the temperature *T* and 0 °C, respectively; *A*, *B*, and *C* are constants. Equation 3 shows that the real-time temperature value can be obtained by the resistance. Three-wire or four-wire systems are generally adopted during actual operation to eliminate the influence of lead resistance. The platinum resistance temperature sensors are often utilized in harsh and high precision-demanding conditions, such as for the internal temperature measurement of the traction motor.

The thermistor temperature sensors are made according to the nature of the semiconductor material resistivity changing with temperature. The resistance value of a commonly employed NTC thermistor decreases with temperature increase, and the relationship in a certain temperature range is expressed as follows [19]:

(4) $$R_{T}=R_{0}e^{B(\frac{1}{T}-\frac{1}{T_{0}})}$$

where *B* indicates the thermistor material constant related to the NTC thermistor materials. The NTC thermistor is often utilized to measure the temperature of the cooling system in the rail transit train.

### 2.4. Application Prospect of the New Signal Sensing Technology

The train operates in an increasingly complex electromagnetic environment with developments focusing on extraordinarily heavy loads and high speed. Traditional electric sensors face these increasingly large difficulties in meeting the demands of electromagnetic interference and insulation. The fiber optic sensor is expected to be applied for rail transit systems due to its electromagnetic interference immunization, high-level voltage insulation, excellent security, and other outstanding advantages [20,21]. The fiber optic sensor is a sensor that uses optical fibers as its sensing material, and the parameters of the light waves can change with the measured signals. Relevant research has been conducted in recent years on sensing signals, such as current, voltage, speed, and temperature measurements by means of optical fibers.

In terms of current sensing, the fiber optical current sensor has become the most potential optionm and has already been applied in power systems [22].

In terms of voltage sensing, the fiber optical voltage sensor uses a non-metallic electro-optic crystal as its sensing element, with working principles based on the Pockels or Kerr effect. The piezoelectric crystal and fiber Bragg grating have been adopted to develop a 13.8 kV voltage sensor with a precision of up to 0.2% [23].

In terms of fiber optic velocity measurements, ordinary photoelectric speed sensors would experience train speed jumping during braking or starting, due to electromagnetic interference [24]. Replacing the photoelectric encoders with fiber optical encoders can solve this issue.

In terms of fiber optic temperature measurements, the platinum resistor temperature sensor has the disadvantage of insufficient insulation. If fluorescence fiber optic temperature sensors [25] with no metal components are used, then the aforementioned problem can be satisfactorily solved.

## 3. Sensor Data Acquisition and Processing 

**Figure 4:**
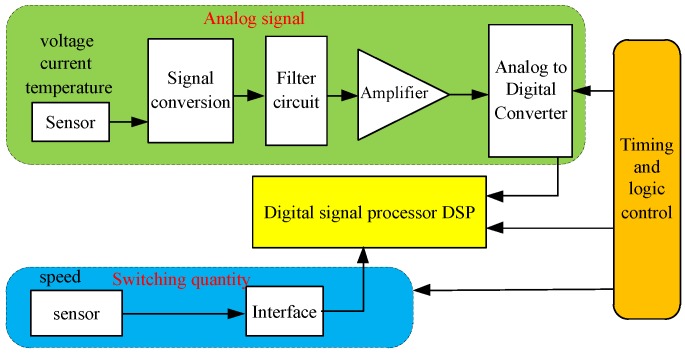
Sensor signal acquisition and processing.

The signals obtained by means of signal sensing technology are diverse, apt to attenuation, and susceptible to interference [26], therefore, acquiring and processing the sensor output signal are also important problems that must be considered. Figure 4 shows that the speed sensor output signal can be acquired and processed via a simple interface circuit; however, processing the analog signal is relatively complicated, and the signal processing requires more steps, such as analog signal processing, analog-to-digital converter (ADC) data sampling, and digital filtering processing. The technology for analog signal processing, data sampling, and digital filtering are summarized in the following section.

### 3.1. Analog Signal Processing

The sensor analog output signals feature different categories (e.g., voltage, current, and impedance types), significant weakness, high output impedance, and vulnerability to interference. The processing of analog signals is commonly made up of parts, such as the current or frequency–voltage conversion, impedance matching, filter, and amplifying circuits, with the purpose to make the sensor output signals adaptive to ADC acquisition. As an important unit of signal processing, the analog filter can restrain noise and attenuate useless frequency signals. Notably, the inverter frequency in the traction system changes with the motor speed, whereas the grid voltage and four-quadrant rectifier input current are fixed at 50 Hz; besides, there are some possibilities with a frequency approximated to zero, such as the DC-link voltage, cooling water temperature, and motor temperature. This condition requires that the filters have different parameters for different data.

**Figure 5:**
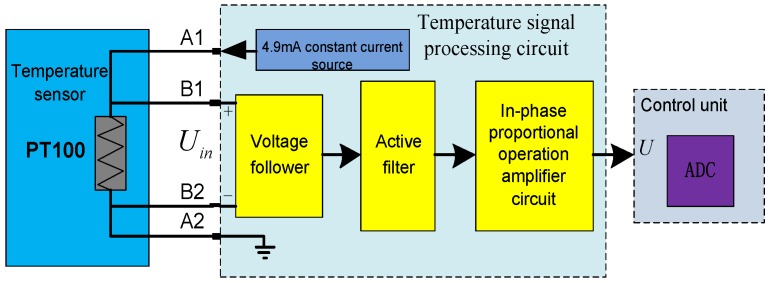
Functional block diagram for temperature signal processing.

Figure 5 shows a typical signal processing circuit of traction control unit temperature sensors. Both ends of PT100 are connected to a 4.9 mA constant current source through A1 and A2. The voltage follower obtains a differential input voltage *U*_in_ through B1 and B2, and generates a low output resistance voltage signal. The output signal enters into the filter circuit and the amplifier circuit for satisfying the ADC requirement.

### 3.2. Sensor Data Sampling Technology

**Figure 6:**
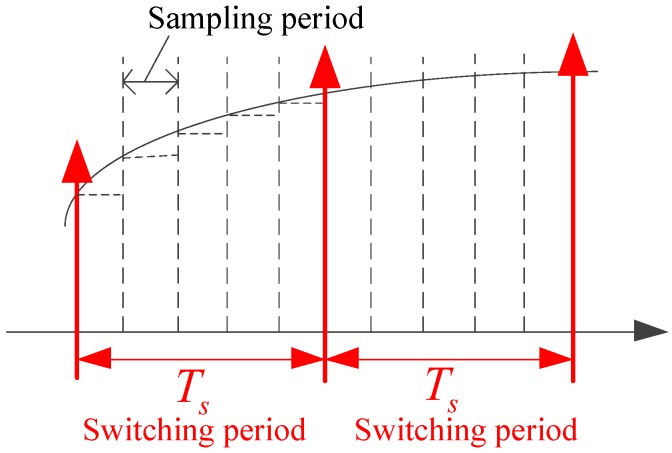
Schematic diagram for sampling in a switching cycle.

Collecting the data at discrete sampling times is necessary within digital control of the traction system. The sampling time of the analog signal must be considered. Shannon sampling theory [27] shows that in order to record all information contained in a ω-bandlimited continuous signal, it suffices to record samples of the signal’s amplitude at discrete points in time, namely at the so-called Nyquist rate of 2*ω* samples per second. When the switching frequency is comparatively high, it can be approximately assumed that the sampling frequency needs to be twice that of the switching frequency. However, time delays gradually become apparent and therefore present a significant effect on the performance of the control system in the case of a low switching frequency. Figure 6 shows that five rounds of data acquisition are completed in one switching cycle, thereby making the control and modulation sampling time independent.

The effects of sampling time on inverter switching loss, harmonic loss, and torque ripple of a direct torque-controlled induction motor drive are investigated in [28]. A cost function that consists of motor harmonic losses, torque ripples, and inverter switching losses, are defined to determine the optimum value of sampling time. The sampling time is selected based on minimizing this cost function. Reference [29] studies the effect of sampling time, switching frequency, and quantization step on the position performance of a vector-controlled inverter, through experiments performed. In the digital control system, a single sampling method is somewhat limited under certain conditions, and researchers have paid much attention to multiple sampling theory. The digital control system that boasts multiple sampling rates can realize different control functions that the single-sampling-rate digital control system hardly attains, such as simultaneous stabilization, strong stabilization, and system robustness improvement [30,31]. A multi-rate model reference adaptive system (MRAS) is proposed in [32] to estimate the rotor time constant, and to obtain a highly stable and less noisy estimation result for the rotor time constant for the asynchronous motor. Experimental results has verified the effectiveness of this algorithm. The Kalman filtering algorithm is combined with multiple sampling in [33] to estimate the permanent magnet synchronous motors speed. A slow sampling frequency is adopted for the motor current, whereas a fast sampling frequency is adopted for the output voltage. Simulation and real-time experiments are conducted to verify that its algorithm is superior to that of the single-sampling-rate Kalman filtering algorithm, in terms of recognition accuracy and convergence stability. Multi-rate theory, which adopts different sampling frequencies in the speed loop, flux loop, and parameter estimation to lower the torque ripple in direct torque control systems of asynchronous motors, is also utilized in [34,35].

### 3.3. Digital Filtering Technology

The digital signals converted from the ADC or digital interface still cannot be utilized directly. They may also require further processing, such as digital filtering. Digital filtering is widely applied in train traction systems, such as in the following:

1. Digital filtering can be used as an effective supplement to hardware filtering, and can overcome the disadvantage of fixed hardware filtering frequency characteristics.
2. Digital filtering can be employed to filter indirect measure signals. For example, motor torque signals are observed by flux linkage and current signals. The observed torque has significant high-frequency fluctuations, and cannot be utilized to the control loop before filtering.
3. The digital filter is also used to extract particular signals. For example, the DC link voltage of the traction drive system has twice the power frequency pulsation. A notch filter is required to extract the ripple voltage. When the high-frequency voltage is injected to observe the location of the permanent magnet synchronous motor rotor, a band-pass or band-stop filter is also required to extract certain signals [36,37].

Frequency response characteristics show that digital filters can be divided into four types: low-pass, high-pass, band-pass, and band-stop filters. They also can be divided into finite impulse response (FIR) and infinite impulse response (IIR) filters [38], according to the length of the unit impulse response *h*(*n*).

The unit impulse response *h*(*n*) of the FIR filter is a length-finite sequence, and the time-domain input and output relation can be expressed as follows:

(5) $$y(n)=\sum_{i=0}^{N-1}h(i)x(n-i)$$

where *N* indicates the number of the FIR filter taps, *h*(*i*) indicates the tap coefficient at level *I* (unit pulse response), *x*(*n* − *i*) indicates the input signal after a delay of *i* taps, and *y*(*n*) indicates the output signal at the moment *n*. The time-domain input and output relation of the IIR filter can be expressed as follows:

(6) $$y(n)=\sum_{r=0}^{M}b_{r}x(n-r)+\sum_{k=1}^{N}a_{k}y(n-k)$$

where *b_r_* and *a_k_* are constants. Equations (5) and (6) show that the FIR filter output is only related to the input, but not directly related to the previous output. Thus, its network structure contains no feedback branch. The IIR filter output consists of two parts. The first part is an input M-section delay chain structure with all delay taps weighted and added up, whereas the second part is the sum of the *y*(*n*) delay taps after being weighed, so it contains a feedback network. The transfer function of the IIR filter system can have its poles located anywhere within the unit circle, such that a low-order filter can offer higher frequency selectivity compared with the FIR filter, thus the IIR filter can achieve higher economy and efficiency than FIR [39]. Therefore, the IIR filters are more widely adopted in traction systems compared with the FIR filters. For example, the grid voltage, torque, and current filters are generally realized by the first- or second-order low-pass filters in the form of IIR. Reference [40] proposed a fault diagnosis method of DC-link circuit based on a notch filter in an electrical power traction converter. The notch filter is equivalent to a band stop filter and can be used to eliminate the selected frequency component without influencing the other frequency components of the system. The second order notch filter has a transfer function, which is expressed as follows:

(7) $$y=\frac{s^{2}+\omega_{2}^{2}}{s^{2}+2\tau \omega_{2}s+\omega_{2}^{2}},$$

where *ω*_2_ indicates the notch frequency, and τ indicates the parameter related to the quality factor. It can be converted to the *z*-plane from s-plane using the impulse response invariant method, or the bilinear transformation method. For example, the bilinear transformation $s=2f_{s}\frac{1-z^{-1}}{1+z^{-1}}$ and the transfer function of the notch can be easily converted to a digital wave trap, as shown in the following:

(8) $$y[k]=a_{0}x[k]+a_{1}x[k-1]+a_{2}x[k-2]-b_{1}y[k-1]-b_{2}y[k-2],$$

where *a*_0_, *a*_1_, *a*_2_, *b*_1_, and *b*_2_ are constants. *x*[*k*] and *y*[*k*] indicate the input signal and filter response, respectively.

## 4. Sensor Fault Diagnosis

The sensor signals after acquisition and processing generally enter the train monitoring, control, and other relevant systems for monitoring, or a closed loop control. The reliability of the sensor signals must be ensured in these conditions. Any error signal fed into the train control system without processing can result in serious consequences; thus, diagnosing train sensor faults is necessary for safety and economy. Sensor fault diagnosis currently adopts hardware and analytical redundancy methods [41]. The hardware redundancy method employs multiple sensors to measure the same system parameter, thereby increasing system complexity and costs. The analytical redundancy method mainly provides redundancy information through analytical relations between different output values of the system. It can be divided into two methods, which are dependent on, or independent of models. In this section, only analytical redundancy methods are presented.

### 4.1. Current Sensor Fault Diagnosis

The current sensor is the basis for controlling the traction inverter. The majority of existing fault diagnosis studies are based on the control model of asynchronous or permanent magnet synchronous motors. These methods generally design a particular observer and then determine whether the current sensor is faulty, by comparing the observed information with the measured information.

**Figure 7:**
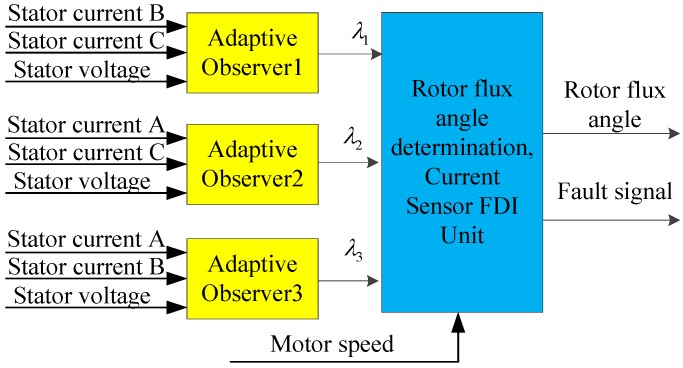
Current sensor fault diagnosis method in [43].

The fault diagnosis method is different between asynchronous motors and permanent magnet synchronous motors, due to their different models. In terms of an asynchronous traction motor, a full-order adaptive observer is adopted in [42] to produce a residual error, and then to judge the current sensor fault according to the residual error and the given threshold value. The fault detect method can isolate the incipient fault of a speed sensor and current sensors in real-time, but the performance of the state observer is degraded at very low speed. In reference [43], three Luenberger observers are used to realize a fault diagnosis of the current sensors, as shown in Figure 7. The proposed three paralleled adaptive observers are capable of current sensor fault detection and localization. An adaptive current observer that can identify the rotor resistance is proposed in [44], with the estimated phase currents and rotor resistance being sent to a decision-making unit, which identifies the faulty sensor type based on a deterministic rule base. Unlike the other proposed model-based fault diagnosis systems using a bank of observers, with the proposed method, only one current observer with rotor-resistance estimation is sufficient for the isolation of all sensors faults. 

In terms of a permanent magnet synchronous motor, a current sensor fault diagnosis module for the harsh working conditions of the permanent magnet synchronous traction motors on trains is designed in [45]. This module adopts sliding mode observers to reconstruct the current sensor fault signals. The module can monitor the possible failures of the system current sensors, and the failure severity and types can also be judged according to the reconstructed fault signals. A sliding mode current observer is designed in αβ coordinates to eliminate the effects of unknown disturbances in [46], then the phase current sensor faults are reconstructed by means of an adaptive method. This method not only can accurately identify and reconstruct intermittent offset faults, slowly varying offset faults and abrupt gain faults in real-time, but also the sliding mode observer has better robustness to inaccurate mathematical models, external disturbances, and parameter perturbation.

These model-based methods for current sensor fault diagnosis share the same problem in that a motor control model is required, and the diagnosis accuracy is related to the accuracy of the motor parameters.

Another current sensor fault diagnosis methods do not require the motor model. A simple and effective method is proposed in [47] for current sensor fault diagnosis according to the basic idea that the phase current sum is zero for three-phase motors. This method only requires the measured phase current，which can not only locate the faulty current sensor, but can also identify the fault modes of the current sensor. A signal-based analysis for the diagnosis of current sensor faults in permanent magnet synchronous motor (PMSM) drives is presented in [48]. Contrary to classical approaches for sensor fault diagnosis, based on residual generation through observers, the signal based uses only three-phase currents to define three diagnostic variables, and is suitable for such occasions where the motor parameters change greatly. The proposed diagnosis method requires low computational resources, and no detection thresholds are needed, since only the signs of the diagnostic variables are used. Neural network and fuzzy control technology are also widely applied in current sensor fault diagnosis. An artificial neural network is used for current sensor fault diagnosis, and the Levenberg–Marquardt algorithm is adopted to train the internal neural network in [49], which considers the motor speed change. A fuzzy-based sensor validation strategy for AC motor drives is proposed in [50], and the major difference between the proposed method and the traditional method is that the thresholds for sensor validation are dynamically generated via uncertainty propagation, rather than being predetermined.

### 4.2. Speed Sensor Fault Diagnosis

**Figure 8:**
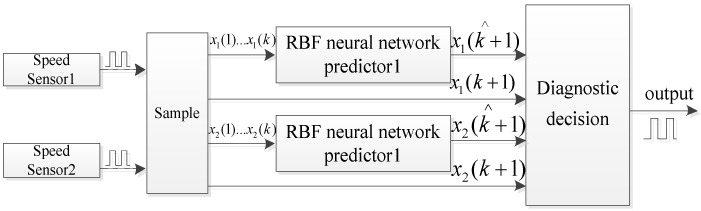
Schematic diagram for speed sensor fault detection based on the neural network algorithm.

In speed sensor fault diagnosis, model-based fault diagnosis methods also rely on the motor control model. They generally judge whether a speed sensor is faulty, by comparing the observer output signal with the actual speed. The observer can be constructed through different methods, such as the extended Kalman filtering method [51], the Luenberger observer [52], and the model reference adaptive observer [53]. The estimated current no longer matches the actual value in fault condition, and this can be easily expanded to speed sensorless control. The main disadvantages of such techniques are that they are complex and dependent on systems parameters. Fault diagnosis methods of speed sensors were compared, based on the MRAS and artificial neural network in asynchronous motors, in [54]. It was concluded that the difference between the detection time for both algorithms was very slight. An online fault detection diagnosis method of two channel speed sensors based on the radial basis function neural network is presented in Figure 8. This method works by selecting the measured output of the speed sensor at the first k moments as the input vectors of the Radial Basis Function neural network predictor, compared to the predicted value of the speed sensor output to at the next moment, carry out fault diagnosis. The advantage of an artificial neural network is that it has parallel distribution processing ability, and can fully approximate complex nonlinear relationships. The disadvantages of neural networks are that they require a large number of parameters, and need long learning time.

### 4.3. Temperature Sensor Fault Diagnosis

Trains suffer from severe vibrations during operation, which can cause temperature sensor faults (e.g., open or short circuits). Thus, detecting temperature sensor faults is necessary. The temperature model is complex, such that the fault diagnosis methods are mainly independent from the models. Temperature sensor faults are generally classified into two types. The first type is a sensor with saturation output. These faults can be rapidly identified, but their true temperature value is difficult to reconstruct. The second fault still has inexact measured outputs that change with the temperature as shown in Figure 5. The A2 or B2-open fault only means that the measured value will deviate from the normal value. At this point, the real temperature in such fault conditions can be obtained by means of data fitting to reconstruct real temperature values.

## 5. Intelligent Sensor Technology

The development of intelligence, networking, and automation of rail transit systems has required the use of sensors with high precision and reliability in the field. Data processing capabilities such as self-inspection, self-calibration, and self-compensation are also necessary for sensors. Traditional sensors cannot meet these requirements, whereas intelligent sensors [55] with microprocessors and integrated sensors can process the input data and transmit the relevant information to external users. Intelligent sensors represent the development direction of the next generation of sensors.

### 5.1. Technical Features of Intelligent Sensors

**Figure 9:**
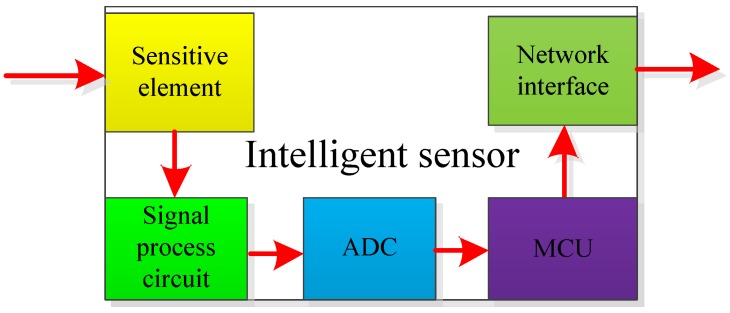
Structure chart of intelligent sensors.

The intelligent sensor has a built-in microprocessor, and its structure is shown in Figure 9. Its working mode changes from passive measure to active measure, unlike traditional sensors. The sensor also has two technology features. The first feature is that it has data processing capacities, such as self-diagnosis, self-correction, self-learning, and self-adaption [56]. Its second feature is that it has a network interface [57].

An intelligent sensor can implement the conversion between analog/digital quantities and complete acquisition and processing, by virtue of its built-in AD converter and microprocessor. In particular, digital filtering, nonlinear compensation, zero drift, and temperature compensation, as well as self-diagnosis and self-protection, are implemented. The embedded microprocessor structure of an intelligent sensor can also provide it with features of advanced function. Thus, advanced algorithms can be adopted such that the sensor can learn, conduct fault diagnosis, and reconstruct the measured signal according to a certain behavioral standard. These capabilities largely improve sensor reliability and measurement accuracy.

Networking is the second main feature of intelligent sensors, which can use standardized bus or wireless network interfaces to establish communication among intelligent sensors, and between the intelligent sensors and main controller. Therefore, this not only can implement the digitalization of transmission signals, but also can easily expand the system. With the development of network-based sensors, intelligent sensors data have no spatial limit. Hence, manufacturers can diagnose sensor faults in a timely manner and guide users to immediate troubleshooting.

### 5.2. Key Technology of Intelligent Sensors

The key technology of intelligent sensors according to the signal flow order can be analyzed in three aspects: signal sensing and integration technology, signal processing technology, and signal transmission technology.

#### 5.2.1. Signal Sensing and Integration Technology

Intelligent sensors can be produced by integrating sensitive elements, a signal processing circuit, and a micro-processing unit on a chip with silicon material based on micro- electromechanical system (MEMS) technology, and large scale integrated circuit technology [58]. In [59], a software-programmable mixed-signal embedded platform for intelligent sensor interfacing with limited complexity, suitable for automotive applications, has been proposed. It has a cost-effective design of fusion algorithms, and relevant prototyping implementation on a mixed-signal acquisition hardware platform, including a configurable analog front end, analog-to-digital converters, on-chip digital processing, and communication resources. The development of multi-sensor integration technology has also enabled the integration of multi-sensitive elements on the same chip to attain a comprehensive measure. This integrated sensor has the advantages of smaller volume, lower cost and higher reliability, and all the sensor data processing function can be implemented in this new sensor.

#### 5.2.2. Signal Processing Technology 

Signal processing technology can be improved from two aspects of chip design and algorithm design. In terms of intelligent sensor design, a novel design platform, called SensASIP, for sensor conditioning devices, suitable for automotive applications, is proposed in [60] in order to reduce the cost and static power consumption of traditional application specific integrated circuit (ASIC) sensors. It is a design platform targeting a microprocessor architecture enhanced by dedicated instructions for computing intensive sensor signal processing tasks. If the sensor integrates self-diagnostic circuit, allowing detecting fault such as over-current and over temperature to CPU by means of a digital interface, sensor fault can be detected immediately without the complicated algorithm [61]. In terms of intelligent sensor algorithm design, an intelligent sensor can integrate all types of intelligent algorithms in a micro-processor unit and accomplish its special functions [62,63]. For example, if the artificial intelligence technology is built in the sensor, then it can directly accomplish fault diagnosis and predict the key components in a train (such as the traction converter, traction transformer, traction motor, and pantograph). 

#### 5.2.3. Signal Transmission Technology

Intelligent sensor signal transmission is the essential functions. For example, the safety and reliability are critical for the railway applications, so the predictive diagnosis for traction system is an important means for safety and reliability. Predictive diagnosis of high-power transformer faults by networking vibration measuring nodes is proposed in [64,65], where vibration-based mechanical stress diagnosis is implemented together with electrical (voltage, current, impedance) and thermal degradation analysis. The diagnostic system is achieved through a distributed network of measuring nodes. 

Wireless signal transmission is also a research hotspot in current intelligent sensors [66]. In recent years, the wireless sensor network technology has been rapidly developed [67]. Wireless sensor network integrates sensor technology, wireless communication technology and distributed information processing technology and so on. The research direction focuses on different aspects, such as compatibility to any other network or interface, security and low power consumption. The security requirements of wireless sensor networks are based on their own conditions, which is the biggest difference from the general wireless communication networks. The research of wireless sensor network security mainly includes the various threats that sensor networks are confronted, the schemes of pairwise key management in sensor networks, the protocol of encryption for security [68]. There are few researches on the security of train sensor networks, but the research in other fields has already been carried out. Security issues of a home area network which is the network of communicating loads, sensors, and appliances within the customer’s premises for smart grid have been discussed in [69]. Some solutions are proposed in order to define a high-level architecture implementing privacy and security techniques in the grid.

## 6. Future Research Hotspots and Challenges on Railway Transit Sensor

With the development of high speed and heavy duty train, the electromagnetic environment is becoming more and more complicated. The traditional electrical type sensors are becoming difficult to meet the demand of electromagnetic interference and insulation performance. Therefore, researchers have paid more attention to the new sensors in rail transit, such as the optical fiber sensors. The optical fiber sensor has good electromagnetic interference and insulation performance, suitable for measuring the temperature and the speed of the train. Fiber grating sensor has the advantages of high sensitivity, small size, the ability to contact network, so it can be used for health monitoring of train, bridges and tunnels. There are still some issues that need to be dealt with, such as feature extraction and demodulation, and cost reduction.

The intelligence, integration and digitalization of sensors are the important technology for the safety of the railway traffic equipment, and it also the key technology of rail transit equipment upgrade. Using new materials and new structures to develop integration, intelligence, low power consumption and high reliability sensors is the hotspot and trend of the research. With the continuous development of ASIC chip design technology, MEMS technology and the intelligent material technology, the intelligent sensor will be further improved.

Rail transit trains form a large and complex system, which requires the coordinated work of all subsystems. Wireless sensor network is composed of a large number of sensor nodes, which can quickly form an adaptive network topology for cooperative sensing and processing of distributed dynamic information. Through wireless sensor networks, the decentralization subsystem signal data can become interconnected and coordinated. Network architecture, routing algorithms, data fusion, data security, and other key technologies need to be further researched.

## 7. Conclusions

The efficient, safe, and intelligent running of rail transit trains cannot be separated from the comprehensive sensing of the entire system. Large numbers of different sensor types will be applied on trains in the future. However, train onboard space is limited, and the working environment is relatively complicated. Future sensors must have the following technical features to meet the aforementioned demands: utilization of microchip-based sensors to break through space constraints, employment of compound sensors to attain efficient sensing and high-cost performance, and adoption of wireless digital sensors to accomplish the efficient transmission of sensor data. Miniaturized, multi-functional, intelligent, and networked sensors are the trend of future sensor developments.

## References

[1] Zhao N., Roberts C., Hillmansen S., Nicholson G. (2015). A Multiple Train Trajectory Optimization to Minimize Energy Consumption and Delay. IEEE Trans. Intell. Transp. Syst..

[2] Zhang H., Quan W., Song J., Jiang Z., Yu S. (2016). Link State Prediction-Based Reliable Transmission for High-Speed Railway Networks. IEEE Trans. Veh. Technol..

[3] Dong H., Ning B., Chen Y., Sun X., Wen D., Hu Y., Ouyang R. (2013). Emergency Management of Urban Rail Transportation Based on Parallel Systems. IEEE Trans. Intell. Transp. Syst..

[4] Glickenstein H. (2011). China Becomes a Major Player in the Rail Market. IEEE Trans. Veh. Technol..

[5] Quraan M., Siam J. Modeling and Simulation of Railway Electric Traction with Vector Control Drive. Proceedings of the 2016 IEEE International Conference on Intelligent Rail Transportation (ICIRT).

[6] Aguirre M., Calleja C., Lopez-de-Heredia A., Poza J., Aranburu A., Nieva T. FOC and DTC comparison in PMSM for railway traction application. Proceedings of the 2011 14th European Conference on Power Electronics and Applications.

[7] Hu H., Li Y.D., Zeng Y. Direct Torque Control of Induction Motor for Railway Traction in Whole Speed Range. Proceedings of the IEEE 2002 28th Annual Conference of the Industrial Electronics Society, IECON 02.

[8] Yin J., Chen D., Li L. (2014). Intelligent Train Operation Algorithms for Subway by Expert System and Reinforcement Learning. IEEE Trans. Intell. Transp. Syst..

[9] Hengyu L., Hongze X. An integrated intelligent control algorithm for High-Speed Train ATO systems based on running conditions. Proceedings of the Third International Conference on Digital Manufacturing and Automation (ICDMA).

[10] Zhongguancun’s Internet of Things (IOT) Industry Alliance (2011). Beijing-Shanghai high-speed railway security guard—1000 intelligent safety sensors. Int. Things Technol..

[11] Liran W., Mingli W., Kejian S. Analysis of Electrical Load Characteristics of HXD1B locomotive based on measured data. Proceedings of the 2014 IEEE Conference and Expo Transportation Electrification Asia-Pacific (ITEC Asia-Pacific).

[12] Gou B., Ge X., Wang S., Feng X., Kuo J.B., Habetler T.G. (2016). An Open-Switch Fault Diagnosis Method for Single-Phase PWM Rectifier Using a Model-Based Approach in High-Speed Railway Electrical Traction Drive System. IEEE Trans. Power Electron..

[13] Youssef M.Z., Woronowicz K., Aditya K., Azeez N.A., Williamson S.S. (2016). Design and Development of an Efficient Multilevel DC/AC Traction Inverter for Railway Transportation Electrification. IEEE Trans. Power Electron..

[14] Kumar A., John V. Power Electronic Converter for Characterization of Hall-effect Current Sensors. Proceedings of the 2014 IEEE International Conference on Power Electronics, Drives and Energy Systems (PEDES).

[15] Jobling D. New Open-Loop Current Transducers with near Closed-Loop Performance. Proceedings of the International Exhibition and Conference for Power Electronics.

[16] Lorenzen D.M., Falls B. (1991). Rotary Speed Sensor with Base line Compensation of Hall Cell Output Signal. U.S. Patent.

[17] Xu Z., Wang W., Sun Y. (2012). Performance Degradation Monitoring for Onboard Speed Sensors of Trains. IEEE Trans. Intell. Transp. Syst..

[18] Liu J., Li Y., Zhao H. A Temperature Measurement System Based on PT100. Proceedings of the International Conference on Electrical and Control Engineering (ICECE).

[19] Narayana K.V.L., Kumar V.N. (2016). Development of an Intelligent Temperature Transducer. IEEE Sens. J..

[20] Chen X., He S., Li D., Wang K., Fan Y., Wu S. (2016). Optical Fiber Voltage Sensor Based on Michelson Interferometer Using Phase Generated Carrier Demodulation Algorithm. IEEE Sens. J..

[21] Zhang Z., Yan L., Pan W., Luo B., Wang P., Guo L., Zhou W. (2012). Sensitivity Enhancement of Strain Sensing Utilizing a Differential Pair of Fiber Bragg Gratings. Sensor.

[22] Zheng Y., Wang X., Meng H. Study on the characteristics of all fibre optical current sensor linear fitting algorithm. Proceedings of the IEEE International Conference on Electronic Measurement & Instruments (ICEMI).

[23] Allil R.C.d.B., Werneck M.M. (2011). Optical High-Voltage Sensor Based on Fiber Bragg Grating and PZT Piezoelectric Ceramics. IEEE Trans. Instrum. Meas..

[24] The Ministry of Railways Transport (2010). Evaluation Opinions about the Solutions of CRH LKJ Speed Signal Problems.

[25] Hu C., Wang Y. Digital signal processing for fluorescence-based fiber-optic temperature sensor. Proceedings of the Advanced Sensor Systems and Applications.

[26] Maya-Hernandez P.M., Alvarez-Simon L.C., Sanz-Pascual M.T., Calvo-Lopez B. (2014). An Integrated Low-Power Lock-In Amplifier and Its Application to Gas Detection. Sensors.

[27] Hao Y., Kempf A. On a Non-Fourier Generalization of Shannon Sampling Theory. Proceedings of the 10th Canadian Workshop on Information Theory (CWIT).

[28] Kaboli S.H., Zolghadri M.R., Homaifar A. (2003). Effects of Sampling Time on the Performance of Direct Torque Controlled Induction Motor Drive. IEEE Int. Symp. Ind. Electron..

[29] Aphiratsakun N., Techakittiroj K., Threevithayanon W. Vector-control of induction motor: Study of sampling time, switching frequency and output quantization step. Proceedings of the 2004 IEEE Region 10 Conference TENCON 2004.

[30] Lin B., Recke B., Knudsen J.K.H., Jørgensen S.B. (2009). Data-driven soft sensor design with multiple-rate sampled data: A comparative study. Ind. Eng. Chem. Res..

[31] Yamaguchi N., Hasegawa M., Doki S., Okuma S. (2006). New approach for stability improvement of speed sensorless induction-motor controls at zero frequency using multirate adaptive observer. IEE Proc. Electr. Power Appl..

[32] Wang S., Dinavahi V., Xiao J. (2013). Multi-rate real-time model-based parameter estimation and state identification for induction motors. IET Electr. Power Appl..

[33] Xu P., Xiao J., Wang S., Lu K. (2014). Application of multirate Extended Kalman Filter to speed estimation of PMSM. Comput. Eng. Appl..

[34] Pandya S.N., Chatterjee J.K. Torque Ripple Minimization in Direct Torque Control Based Induction Motor Drive Using Optimal Multirate Sampling Technique. Proceedings of the 2010 Joint International Conference on Power Electronics, Drives and Energy Systems & 2010 Power India.

[35] Pandya S.N., Chatterjee J.K. Torque Ripple Minimization in Direct Torque Control based IM Drive Part-II: Multirate Control Strategy. Proceedings of the 2008 Joint International Conference on Power System Technology and IEEE Power India Conference.

[36] Ramezani M., Ojo O. (2016). The Modeling and Position-Sensorless Estimation Technique for A Nine-Phase Interior Permanent-Magnet Machine Using High-Frequency Injections. IEEE Trans. Ind. Appl..

[37] Mohammed O.A., Khan A.A., El-Tallawy A.M., Nejadpak A., Roberts M.J. (2012). A Wavelet Filtering Scheme for Noise and Vibration Reduction in High-frequency Signal Injection-Based Sensorless Control of PMSM at Low Speed. IEEE Trans. Energy Convers..

[38] Beylkin G., Lewis R.D., Monzón L. (2012). On the Design of Highly Accurate and Efficient IIR and FIR Filters. IEEE Trans. Signal Process..

[39] Halikias G.D., Jaimoukha I.M. Design of Infinite Impulse Response (IIR) filters with almost linear phase characteristics. Proceedings of the European Control Conference (ECC).

[40] Gou B., Ge X., Pu J., Feng X. A Fault Diagnosis and Fault tolerant Control Method for DC-link Circuit Using Notch Filter in Power Traction Converter. Proceedings of the 2016 IEEE 8th International Power Electronics and Motion Control Conference (IPEMC-ECCE Asia).

[41] Gao Z., Cecati C., Ding S.X. (2015). A survey of fault diagnosis and fault-tolerant techniques-partⅠ: Fault diagnosis with model-based and signal-based approaches. IEEE Trans. Ind. Electron..

[42] Lee K.S., Ryu J.S. (2003). Instrument fault detection and compensation scheme for direct torque controlled induction motor drives. IEE Proc. Control Theory Appl..

[43] Yu Y., Wang Z., Xu D. (2014). Speed and current sensor fault detection and isolation based on adaptive observers for induction motor drivers. J. Power Electron..

[44] Najafabadi T.A., Salmasi F.R., Maralani P.J. (2011). Detection and Isolation of Speed-, Dc-link Voltage-, and Current-Sensor Faults Based on an Adaptive Observer in Induction-Motor Drives. IEEE Trans. Ind. Electron..

[45] Zhang C.F., Liao H.J., Li X.F., Sun J., He J. (2016). Fault Reconstruction Based on Sliding Mode Observer for Current Sensors of PMSM. J. Sens..

[46] Huang G., Luo Y.P., Zhang C.F., He J., Huang Y.S. (2016). Current sensor fault reconstruction for PMSM drives. Sensors.

[47] Gou B., Ge X., Liu Y., Feng X. (2016). Load-current-based current sensor fault diagnosis and tolerant control scheme for traction inverters. Electron. Lett..

[48] El Khil S.K., Jlassi I., Estima J.O., Mrabet-Bellaaj N., Cardoso A.J.M. (2016). Current sensor fault detection and isolation method for PMSM drives, using average normalised currents. Electron. Lett..

[49] Klimkowski K., Dybkowski M. Neural Network Approach for Stator Current Sensor Fault Detection and Isolation for Vector Controlled Induction Motor Drive. Proceedings of the 2016 IEEE International Power Electronics and Motion Control Conference (PEMC).

[50] Li H., Monti A., Ponci F. (2012). A Fuzzy-Based Sensor Validation Strategy for AC Motor Drives. IEEE Trans. Ind. Inform..

[51] Zhang X., Foo G., Vilathgamuwa M.D., Tseng K.J., Bhangu B.S., Gajanayake C. (2013). Sensor fault detection, isolation and system reconfiguration based on extended Kalman filter for induction motor drives. IET Electr. Power Appl..

[52] Bourogaoui M., Jlassi I., Khil S.K.E.L., Sethom H.B.A. An Effective Encoder Fault Detection in PMSM Drives at Different Speed Ranges. Proceedings of the 2015 IEEE 10th International Symposium on Diagnostics for Electrical Machines, Power Electronics and Drives (SDEMPED).

[53] Chakraborty C., Verma V. (2015). Speed and Current Sensor Fault Detection and Isolation Technique for Induction Motor Drive Using Axes Transformation. IEEE Trans. Ind. Electron..

[54] Klimkowski K., Dybkowski M. A Comparative Analysis of the Chosen Speed Sensor Faults Detectors for Induction Motor Drives. Proceedings of the 2015 International Conference on Electrical Drives and Power Electronics (EDPE).

[55] Sergeyev I.Y. Signal processing system of intelligent sensor with nonlinear characteristic. Proceedings of the 2013 IEEE 2nd International Conference Actual Problems of Unmanned Air Vehicles Developments (APUAVD).

[56] Zhang Y., Gu Y., Vlatkovic V., Wang X. Progress of Smart Sensor and Smart Sensor Networks. Proceedings of the Fifth World Congress on Intelligent Control and Automation, WCICA 2004.

[57] Al-Ali A.R., Aji Y.R., Othman H.F., Fakhreddin F.T. Wireless smart sensors networks overview. Proceedings of the Second IFIP International Conference on Wireless and Optical Communications Networks, WOCN 2005.

[58] Gervais-Ducouret S. Next smart sensors generation. Proceedings of the 2011 IEEE Sensors Applications Symposium (SAS).

[59] Saponara S., Petri E., Fanucci L., Terreni P. (2011). Sensor Modeling, Low-Complexity Fusion Algorithms, and Mixed-Signal IC Prototyping for Gas Measures in Low-Emission Vehicles. IEEE Trans. Instrum. Meas..

[60] Sisto A., Pilato L., Serventi R., Saponara S., Fanucci L. (2016). Application specific instruction set processor for sensor conditioning in automotive applications. Microprocess. Microsyst..

[61] Costantino N., Serventi R., Tinfena F., D’Abramo P., Chassard P., Tisserand P., Saponara S., Fanucci L. (2011). Design and Test of an HV-CMOS Intelligent Power Switch With Integrated Protections and Self-Diagnostic for Harsh Automotive Applications. IEEE Trans. Ind. Electron..

[62] Miseje M., Sturcel J. (2003). Signal Processing in Smart Sensor Systems. J. Electr. Eng..

[63] Garcia G.J., Jara C.A., Pomares J., Alabdo A., Poggi L.M., Torres F. (2014). A Survey on FPGA-Based Sensor systems: Towards Intelligent and Reconfigurable Low-Power Sensors for Computer Vision, Control and Signal Processing. Sensors.

[64] Saponara S., Fanucci L., Bernardo F., Falciani A. (2016). Predictive Diagnosis of High-Power Transformer Faults by Networking Vibration Measuring Nodes with Integrated Signal Processing. IEEE Trans. Instrum Meas..

[65] Saponara S. (2016). Distributed Measuring System for Predictive Diagnosis of Uninterruptible Power Supplies in Safety-Critical Applications. Energies.

[66] Stanley M., Gervais-Ducouret S., Adams J.T. Intelligent Sensor Hub Benefits for Wireless Sensor Networks. Proceedings of the 2012 IEEE Sensors Applications Symposium (SAS).

[67] Wang H., Ping Z., Shen J., Zhang L. Key Technology of Network Integration for Urban Rail Transit System. Proceedings of the 2014 International Conference on Wireless Communication and Sensor Network.

[68] Yan-min D.U. (2015). Wireless Sensor Network (WSN) safety review. Comput. Eng. Softw..

[69] Saponara S., Bacchillone T. (2012). Network Architecture, Security Issues, and Hardware Implementation of a Home Area Network for Smart Grid. J. Comput. Netw. Commun..

